# Entertainment and fun in the service of survival: Theatre of the People’s Liberation in the battles of Neretva and Sutjeska

**DOI:** 10.1080/07292473.2024.2409537

**Published:** 2024-10-14

**Authors:** Iva Jelušić

**Affiliations:** Christian Michelsen Institute, Bergen, Norway

**Keywords:** Yugoslav Partisans, Theatre of the People’s Liberation, singing, laughter, fun

## Abstract

Using the accounts of the participants in the battles of Neretva and Sutjeska, particularly the members of the Theatre of the People’s Liberation (*Kazalište narodnog oslobođenja*, KNO), this article focuses on ways of having fun as well as its functions among soldiers and civilians who were primarily busy with escaping enemy encirclements. It reveals the range of experiences and the accompanying emotional registers they were exposed to in everyday life while pondering the role of pleasure in the biggest battles of the Second World War in Yugoslavia.

And always, every performance, no matter where it was given, was a celebration for us as well as for the most diverse audience.

A play in war was a festival of peace.^1^

## Introduction

Yugoslav historiography includes seven major Axis military campaigns undertaken against the country’s communist-led Partisan forces during the Second World War.^2^ Each of the campaigns was an extensive manoeuvre that had as its goal the elimination of the Partisan resistance and the pacification of the Yugoslav countries. This article focuses on two of them: the battle of Neretva (January–March 1943)^3^ and the battle of Sutjeska (May–June 1943)^4^ and their immediate aftermath.

The two military campaigns were the largest counter-insurgency operations by the German Wehrmacht during the Second World War and are considered as the turning point of the People’s Liberation Struggle in Yugoslavia.^5^ Importantly, although the units stationed in the territories of Yugoslavia were of substandard quality when compared with *Wehrmacht* units elsewhere,^6^ for the Partisans they were far superior to their other opponents in terms of military strength, skill, and ruthlessness.^7^ The *Wehrmacht* caused them the greatest war losses, particularly during the Neretva and Sutjeska battles, and almost succeeded in destroying them. At the same time, the German field commander Rudolf Lüters, who participated in the planning and execution of both offensives, wrote that he considered the Partisans well organised and led during both military campaigns. In addition, he assessed their combat morale as surprisingly high.^8^ He was not the only one who made such an observation about the Partisan forces.^9^

The reason why this article focuses on the battles of Neretva and Sutjeska to reflect on the experience of the People’s Liberation Struggle from the perspective of fun is the following: they took place one immediately after the other, and for the people who fought in them, they represented physically and emotionally the most difficult period of the war. Further, since the attacking forces primarily targeted the Partisan Supreme Headquarters, many of the same people participated in both battles. During this period, the Theatre of People’s Liberation (*Kazalište narodnog oslobođenja*, KNO)^10^ travelled with the Supreme Headquarters and the accompanying military formations. Because they had the opportunity to experience the battles directly, this part of the KNO’s existence, lasting a little over six months, is useful to explore from the perspective of the members’ work on organising cultural education and entertainment.

In studying this topic, this article mainly focuses on published ego documents written by some members of the KNO,^11^ particularly on their contributions to the maintenance of morale among the participants and supporters of the Partisan resistance. Sources of this type, as is well known, are firmly rooted in the social and cultural practices of the community in which the individual lives and writes, thus making visible various cultural patterns.^12^ Very much in the approach of the history of everyday life, fragments of the memories of individuals who participated in the battles of Neretva and Sutjeska enable at least a partial, albeit fragmented, perspective on ordinary people’s insistence on the creation of spaces free of fear and death, even when they were seemingly surrounded by the constant terror of warfare.

## Fun and the People’s Liberation Struggle

As sociologist Ben Fincham explains, having fun is primarily:

a social phenomenon. It is had with other people or in relation to other people, it is communicated in ways that make sense to others and it relates closely to [their] sense of social identity.^13^

Notably, in the published memoirs relevant for this research, the authors very rarely explicitly mention either entertainment or having fun; both words in Bosnian/Croatian/Serbian languages translate with the same term, *zabava*. The article, therefore, focuses on memories that correspond to the definition of the word *zabava*: a pleasant pastime, an activity that makes it possible to spend time pleasantly, and social events or performances with music and dancing.^14^

The purpose of focusing on instances of entertainment and having fun in the People’s Liberation Struggle is to explore the meanings and functions ascribed to the everyday activities of the members of the KNO. In other words, the article attempts to illustrate the potential of the Partisan theatrical activism and obtain ‘insight into the intensities of sensory experiences of [theatre], as a spectrum of feelings and affects that arise in a concrete space-time environment’.^15^ By delving into the positive, even joyous, memories of some members of the Partisan resistance in Yugoslavia and considering their entanglements with the wartime destruction and suffering, it highlights the purpose of having fun in the most atrocious situations in which the Partisans found themselves during the war. The analysis of ego documents is guided by the question of how fun is experienced in situations of intense pressure and existential suffering.

In this respect, the words of the Slovenian author and Partisan Matej Bor are indicative: ‘Laughter, cheerfulness in the midst of fire and blood! Does it seem immoral to you? Think about tomorrow, and you will understand!’^16^ Even after the end of the war, the same author continued to contemplate the nature of the People’s Liberation Struggle. In the introduction to a reprint of his 1942 collection of poems *Previharimo viharje* (*Let’s weather the storms*) Bor wrote:

The time of our liberation struggle was not great only because in it great things were decided, but also because ordinary people were great in the service of the great cause. […] People became greater than they were before, so much greater as to seem quite incredible, especially when described in an everyday account.^17^

Bor attributed the enthusiasm of the masses during the Partisan struggle to the widespread practice and encouragement of cultural activities: ‘Bread has become less important than spirit, daily chores less important than the historical mission’.^18^ Further describing the role of wartime culture and art during the People’s Liberation Struggle, the art historian Miklavž Komelj explains that it must be considered as one of the major forces in the war and the revolutionary process. He writes inspiringly about the culture becoming a part of war and revolution as ‘something bright and cheerful: like humour full of wild energy of the masses, who are appropriating their cultural heritage with subversion’.^19^

This article builds on Komelj’s assertion by suggesting that it is necessary to move beyond the view of wartime artistic production only as cultural practice. It claims that the sensation of fun that often radiated from the cultural activities of Yugoslav theatre groups such as the KNO was an important aspect of wartime cultural work. Even if theatrical performances, singing, and dancing at times seemed to be nothing more than an amusing pastime and a light diversion, their implications for the so-called morale of the fighting units as well as the morale of the sick and wounded who had nowhere to hide, and the civilians who were caught by chance within the areas of military manoeuvres, were saturated with social and political purpose. They encouraged the strengthening of collective identity and taking pleasure in the process, especially when feelings of defiance and spite were involved, and could sometimes create a sense of diminution of immediate danger. Moreover, they enabled people to replace fear and pain not only with (however fleeting) enjoyment, but also with the feeling that they had a moral right to hope for better.

It is important to note that this article focuses on the described aspect of fun and entertainment because relevant sources indicate that it was most important to the people who found themselves amid the battles of Neretva and Sutjeska. However, organised entertainment during the People’s Liberation Struggle also had a pronounced socio-political purpose. It emphasised political education and mobilisation, the final goal of which was promotion of the establishment of the communist political and social order in the territory of Yugoslavia. In addition, the cultural education it provided was considered important for the New Man and Woman who would inhabit and build that new communist society. I have explored this productive entanglement of political ideas, artistic inspiration, and the necessity of entertaining the audience elsewhere.^20^

This article comprises three main sections. It first focuses on an episode that occurred during the battle of Neretva: the Partisan actors held an acting performance on a night when it was not yet known whether the military units would manage to win a safe way out of the Neretva valley. There were not many such nights in that period, but, even when the theatre groups did not perform, memoirs suggest that the singing continued. The second part focuses on spontaneous moments of socialising among the Partisans, when they took breaks with recitations and singing during the battle of Sutjeska. The last section analyses the immediate aftermath of the two battles and how the members of the KNO recuperated from the traumatic events of the first half of 1943 and how having fun helped in that process.

## ‘The broken theatre’ …

In early 1943, the Yugoslav Partisans faced a critical phase in their struggle for liberation. Allied victories on the African battlefield at the end of 1942 and the possibility of the Allies choosing the Balkans for further military advances prompted the German *Wehrmacht* to launch a winter offensive.^21^ This section centres on the first part of this period, the battle of Neretva. This battle, described by the historian Stevan K. Pawlovitch as ‘the most determined cycle of operations in the Yugoslav lands’,^22^ took place between January and March 1943. Moreover, as Ben H. Shepherd explains, during this mission ‘the combination of ideology and ruthless military necessity which characterised much of Wehrmacht anti-partisan warfare unleashed a degree of brutality exceeded only in the Soviet Union’.^23^ Its goal was to destroy all armed groups not belonging to the Axis, primarily the central command of the Partisan movement and the units around it as well as the Central Committee of the Communist Party, which were then located in the territory of western Bosnia and the neighbouring Croatian regions.^24^ In order to carry out this planned military undertaking, the Axis forces (Germany, Italy, and the Independent State of Croatia) assembled approximately 100,000 soldiers and twelve air squadrons. In addition, around 15,000 Chetniks joined them.^25^ They fought against approximately 20,000 Partisan soldiers.^26^ Importantly, all unarmed men between the ages of fifteen and sixty found in the area of ‘Tito’s State’ were destined for deportation, while the armed ones were to be executed. Displays of brutality against the civilian population were not punished. Despite its ruthlessness, this level of cruelty still fell short of Hitler’s order to execute everyone, including women and children, found in the areas considered as insurgent.^27^

The Fourth Enemy Offensive for the most part unfolded in the rugged terrain of western and south-western Bosnia and Herzegovina.^28^ In the last days of February, following more than a month of fighting, most of the besieged Partisan forces found themselves in a critical position in the Neretva valley because none of the possible withdrawal routes remained open to them. Soldiers in the encirclement fought for strategic locations every day and returned ‘to the bases’ in the valley in the evening. On one such evening, when the Fourth and Fifth Montenegrin Brigades returned from their combat positions, the Partisan actors put on a show for them at the suggestion of the Partisan military commander Sava Kovačević. The actors presented most of the characteristic parts of the so-called Partisan colourful programme to the gathered soldiers, civilians, and some wounded.^29^ On the fire-lit meadow, the actors first recited poetry and sang, then performed two one-act plays. One of them, recalled in the memoirs of several theatre members, was Anton Chekov’s (romantic) comedy *The Proposal* (*Predlozheniye*). The basis of the plot is the visit of a young nobleman, Ivan Vassilevitch Lomov, to the house of his neighbour, Stepan Stepanovitch Chubukov, to ask for his daughter’s hand in marriage, rather shyly and delicately. Instead of a fancy bourgeois salon, that winter night in the Neretva valley, the nobleman Lomov stepped into the meadow decorated with a tent wing stretched over four sticks that posed as a table inside the rich Chubukov’s parlour. The actor playing Lomov and, therefore, the character who stepped onto the scene, was barefoot and shirtless and wore old peasant trousers and a coat without buttons. Likewise, the noble bride-to-be, Natalya Stepanovna, wore patched pants that were too long for her and *opanci*, leather peasant shoes, on her bare feet. Yet she welcomed her suitor with a seductive smile and coquettish chirps: ‘Oh … you look like you are in a parade! Are you getting ready for a ball?’^30^

According to the composer Nikola Hercigonja, during the Fourth and Fifth Offensives, the actors carefully cut the pages of the printed copy of this comedy, as well as most other available paper, to the size of cigarette paper and smoked them with whatever tobacco they had. Because of this, the characters’ lines changed a bit from performance to performance, but the opening was always the same and never once failed to make the audience laugh.^31^ Laughter was not lacking on this night either. Some laughter was certainly intended for the actors on the ‘stage’ who were trying to bring to life *The Proposal*’s lively comic plot. And some was undoubtedly directed at the discrepancy between the text and the scene and, after all, their own reality. As the actor Braslav Borozan explained:

Laughter, laughter was heard among the spectators because they understood the effort of the actors’ drinking tea from imaginary porcelain cups, lifting the tails of their non-existent tailcoats while preparing to sit on non-existent armchairs.^32^

As was the Partisan custom, after the actors finished the programme, the people present continued to socialise and have fun while someone played the accordion. Despite the exhaustion, cold, and hunger, they created an atmosphere of ‘folk festivity’ (*narodno veselje*) by singing folk songs and dancing circle dances. Reportedly, the event ended only at dawn when the soldiers went back to their combat positions.^33^

Why this event was important to its participants is perhaps best expressed in Sava Kovačević’s statement to the actors at the end of the play: ‘My falcons, you are as valuable to me as an entire brigade’.^34^ In other words, even though the performances of the Partisan theatres became part of wartime everyday life fairly early – educator and poet Đuka Kosak explains with affection that the visits of theatre groups to villages often inspired a joyful and festive mood among the inhabitants – to perform a show in such dire circumstances as those of the Neretva battle with the aim of entertaining the troops and civilians can be considered as an ethical act.^35^ A few hours of carefree laughter, song, and dance were possibly the best remedy and preparation for another day of war.

Entertaining the Partisans, the wounded, and civilians through theatrical performances is comparable to the theatre that functioned in besieged Sarajevo during the wars of the 1990s. Theatrologist Zala Dobovšek explains that the theatre activity that emerged during the siege of the city offered people the opportunity to escape into fiction and quiet their sorrows and fears.^36^ Moreover, journalist Senad Pećanin writes that:

theatre was a ‘spiritual support’ […]. It was an engaged resistance in the sense of proving human superiority in relation to the killers who surrounded the place. […] So, they have a cannon, but I have the theatre. This superiority over evil meant an infinite amount to us.^37^

In the same spirit, Haris Pašović not only continued to direct plays and manage the MESS Theatre Festival, but also organised the first edition of the Sarajevo Film Festival at the end of 1993, aptly named ‘Beyond the End of the World’. When filmmakers Johan van der Keuken and Franck Vellenga visited the film festival, they witnessed the desperate living conditions in the city as well as the packed cinemas. Pašović explained to them that:

there are a certain number of things you cannot live without and still be alive. And among them is film. It is the way of dealing with the world and its magic. And people cannot live without magic.^38^

During both wars, theatre and then film became a form of resistance against terror. That is why Braslav Borozan’s sympathetic description of the KNO’s play in the Neretva valley as the ‘broken theatre’ rings true.^39^ Although in circumstances of deprivation, the KNO played some of the most light-hearted make-believe games they had in their repertoire. And by encouraging shared enjoyment and laughter, the actors not only offered temporary respite and uplifted the spirits of the troops and civilians, but also made a stand against the senseless reality surrounding them and reminded those present of their shared humanity.

Around the same time, the Supreme Headquarters made some crucial decisions. In a frontal attack, the Partisans pushed back some of the German military forces towards Gornji Vakuf, north of the Neretva valley. Instead of making an expected follow-up push further away from Neretva, the Partisan units turned back, while at the same time some of the forces established a bridgehead on the river’s eastern bank held by the Chetniks. With the Axis forces looking elsewhere and the Chetniks suffering losses, the Supreme Headquarters ordered the engineers to repair one of the previously blown-up bridges. All means of transport, howitzers, and surplus supplies were thrown into the river, and printing presses and archives were buried. But the Partisan forces, the central hospital, and civilians retreated across the river and continued eastward over the ‘pathless’ Prenj mountain.^40^ Enemy soldiers did not follow the Partisans across the river Neretva, but the air forces did. The first days after the crossing were perhaps most succinctly but also most intensely described by the composer Silvije Bombardelli, who was then with the Ninth Dalmatian Division:

All night we climbed, all night the columns stumbled, and the sick groaned … tired Dalmatians carried up four thousand wounded on their backs.

And in the morning: frozen, slippery snow and, Stukas … Horses torn apart … Stretchers … Hands.

Oh that fury and that mud, that big, bloody muddy wave. … Even today in my dreams I see a face distorted by horror, hands buried in the ground, that in spasms dig a pit with their nails – shelter from death.

All night, morning, and day …^41^

## … And the broken fun

The battle of Neretva, which was the focus of the previous section, took a huge toll on both civilian and Partisan lives. The Axis forces, however, did not achieve the anticipated goals. Preparations for a new campaign, therefore, had already begun two weeks later. It was launched in mid-May and lasted a month. This campaign, known as the battle of Sutjeska, is the focus of the current section.

The Axis military leadership’s plan remained essentially the same as in the case of the previous offensive: to encircle and eliminate the Main Operational Group, that is, approximately 22,000 Partisan soldiers and their leadership, including Josip Broz Tito. The central hospital, with more than three thousand wounded and ill soldiers, also travelled with the Main Operational Group. The Axis forces intended to drive the main Partisan divisions and their Supreme Headquarters towards the naturally isolated and scarcely inhabited area between the canyons of the Tara and Piva rivers and the Durmitor mountain and then destroy them. For the execution of this plan, they rallied more than 120,000 troops (from Germany, Italy, the Independent State of Croatia, and Bulgaria) and over 300 aircraft. In addition, they prepared extensively for the rugged mountainous terrain of eastern Herzegovina and western Montenegro.^42^ According to Đuro Kladarin, political commissar of the Seventh Division of Banija in 1943, the German forces

spared neither men nor materiel, made enormous efforts and overcame such obstacles that even the Partisans stood in awe. They operated in an extraordinarily rugged terrain and dragged their guns through wild ravines and crags where only experienced mountaineers would normally dare to venture.^43^

After two weeks of fighting, in the first days of June, the Partisans found themselves encircled in the Sutjeska valley.

The reaction of the Partisan leadership to the news of the gathering of large numbers of enemy soldiers around them was delayed. Instead of dispersing their forces in time and disappearing before the gathering enemy forces could position themselves in the strategic locations that enabled them to surround the Partisans, the delayed reaction forced the Main Operational Group, the central hospital, and many civilians to undertake a long retreat with ever diminishing chances of breaking through the encirclement.^44^ This is clearly visible in many published remembrances that include the developments around Sutjeska. More than in any other part of the war, the prevailing impression is that the people caught in the enemy encirclement were just running for their lives. In the course of a thirty-seven-hour-long march, one of many during the retreat towards the Sutjeska valley, the actor Vjekoslav Afrić felt like he was walking

for a year already, maybe more; maybe I have always lived on the move and every day the planes came to bomb and machine-gun us. Every day the earth boiled, belched fire, poured stones, and sowed death. The column treaded over death. It trampled death and ran it over. The column grew. It conquered mountain peaks and riverbeds. It fed on the Soul. Its strength was Thought.^45^

During a break, Afrić shared the warmth of the campfire with a Partisan who recognised him and asked to hear the poem about the sixteenth-century peasant rebel Matija Gubec, a staple in the repertoire of the KNO. The actor was happy to oblige and whispered verses directly in the man’s ear so as not to disturb the others around the fire. By one of the other campfires, someone was singing in a low voice. Mostly, however, the people around them slept, some cried in their sleep, some died. No one ate as there was almost no food. The day that followed was very similar to the previous one.^46^

Memoirs about the Yugoslav Partisan war mention countless nights on the edge of existence with only faint traces of life, similar to the one described here. As much as the morale of the Partisan soldiers depended on the artistic performances by the cultural workers, the consoling effect of art was sometimes better communicated in a whisper. There were, however, seemingly just as many nights like the one described by Afrić’s colleague and friend, the actor Joža Rutić. During the same retreat towards Sutjeska, marching under enemy fire for the majority of the time, permission finally came to take a bit of a respite. After dinner, Rutić remembered Nikola Hercigonja pulling out his accordion and starting to sing the famous revolutionary song *Po šumama i gorama* (*Through the woods and mountains*),^47^ which contains, as the author noted, the text ‘Black hordes don’t scare us,/Heroic blood in us boils,/We won’t give away our lands/To be trampled by the fascists’.^48^ Hercigonja himself did not remember who was the first to sing, but he was sure that all present joined in and that ‘in no time the whole mass of tired, hungry, wet Partisans was singing as if in a trance’.^49^

The memories that focus on relaxed joint singing, and sometimes dancing, perhaps best demonstrate the affective potential of music, that is, its ability to nudge people effortlessly into socialising that in turn demonstrates ‘that amid so much fear and death there was a great deal of life’.^50^ It is appropriate to mention here, for instance, an anecdote related to the retreat to Neretva and the night when the KNO members and some of the Partisan soldiers rested in the village of Bukovica. The KNO’s performance there was interrupted by the incursion of a Chetnik unit. As expected, the performance was stopped, the fighters immediately launched a counter-attack, and the civilians dispersed. It did not take long, however, for the Chetniks, who probably did not expect a swift armed reaction, to run away. Seemingly, no one was in the mood for a theatrical play anymore. Instead, the members of the KNO and the Partisans who had returned from their chase sat around the centre of the village and simply started singing. The local population soon joined them, and, reportedly, together they sang until late into the night. First, they sang revolutionary songs, and when they had gone through every one they knew, they moved on to folk songs originating in the different regions of Yugoslavia until they had exhausted their knowledge and good spirits.^51^

In addition, it was in the context of the retreat to Sutjeska that Hercigonja reflected in a little more detail on the nature and function of ‘singing like this or having entire shows around the campfire’, which, according to his thinking, were festive and intimate events at the same time.^52^ Then, in Rutić’s view, there existed a hope that maybe even the enemy soldiers could hear them singing.^53^ In that case, they should be able to understand the underlying message: that the Partisans they were pursuing and mercilessly killing were neither dead nor defeated. In other words, remembrances such as these describe a bit of socialising and having fun that both Partisans and civilians alike participated in to dissipate their own fear and anguish, but also, especially in the circumstances of the battle of Sutjeska, to express their spite and defiance and to build up their strength. A comparable sentiment was described by folklorist Graham Seal, who researched trench newspapers of English-speaking soldiers during the first world war. Writing about the military poems and songs of ‘those who feared, with considerable justification, that they were very likely about to die’, he very illustratively asserts that their songs ‘present a defiant, communal finger to the likelihood of death or injury inside the machinations of the machinery of war’.^54^ To echo Braslav Borozan’s thoughts, through this broken kind of having fun, they sang in order to prove that they were still there and would continue their resistance the next day as well.

The Partisans clashed ferociously with the Axis forces on the Sutjeska bridgehead from the beginning of June. Fierce battles for strategic high points were fought for several days. Finally, one of the Partisan groups, disobeying Tito’s direct commands, managed to break out northward on 10 June.^55^ Shortly after creating a passage for the transfer of everyone within the encirclement who could manage the escape, ‘a large guerilla [sic] force made it across the road and disappeared into the mountains beyond’.^56^ Three brigades and the majority of the central hospital, comprising over 2000 wounded and largely unarmed medical personnel, remained within the encirclement. Some individuals and isolated smaller groups managed to sneak out of the hostile region, but most of the remaining Partisans, as well as the wounded and civilians, were executed in the ensuing clean-up operation, while a smaller number were deported to Germany, mostly to camps. For the Partisans, it was barely a Pyrrhic victory. During this battle, the Main Operational Group suffered the greatest losses; a third of the Partisans were killed during the battle, and another third were wounded. Tito was among the wounded, which had a negative effect on the morale of the Partisans and their supporters. They had, however, survived and remained a cohesive force, so their struggle continued.

## After Neretva and Sutjeska – fixing the broken

Shortly after crossing over the river Neretva, the writer Veselin Masleša reportedly said to his friends in the KNO: ‘Well, comrades, we have entered history … but which of us will emerge from it?!’^57^ Masleša drowned in Sutjeska a few months later. Most of his comrades, however, survived both battles which have been the focus of the article so far. After escaping the enemy at Sutjeska, the members of the KNO moved slowly northward and took refuge in the village of Seona (eastern Bosnia and Herzegovina, approximately two hundred kilometres north of the site of the Sutjeska battle). They spent several weeks there and then continued westward towards the city of Jajce.^58^ At that time they developed some coping methods based on practising and having fun with art. The written recollections of these coping mechanisms by some members of the KNO are analysed in this section.

Recalling that period, Bombardelli’s wife, the actress Mira Banjanin, recorded how the two of them hid from a sudden bombing in a post office building in a Bosnian town, and how its director invited them to join him for lunch with his family. Noticing the cleanliness and tidiness of their apartment and the hearty lunch on the table, Banjanin commented: ‘The two of us are lousy, dirty, torn, skinny. We introduce ourselves: intellectuals, artists, Partisans … It sounds quite funny and incredible … I don’t think they believed us’.^59^ How scruffy the entire group looked is perhaps best witnessed by the fact that when they arrived in front of the walls of Jajce, one of the members of the KPJ Politburo saw them and ordered that they be allowed into the city only after dark – so that as few people as possible could see them.^60^ Here, the physical appearance of the KNO group is also indicative of their psychological and emotional state. Namely, while they actively returned to their work of, as they phrased it, bringing culture to the masses, they were trying to come to terms with what they had been through in the previous few months. Their internal struggles were visible in the way they performed their shows. Instead of the habitual insistence on artistic excellence, there was more nonchalance, carelessness, and even conflict situations on stage.

For instance, the KNO performed *The Proposal* in Seona as well. During the first performance, the actor who played the young nobleman Lomov, then wearing shoes and with a bouquet of field flowers in his hands, came to ask for the hand of Chubukov’s daughter just as he should do. Instead of keeping the character’s shy and delicate demeanour, however, the actor who played him and the man who played Chubukov spontaneously burst into a sincere quarrel, scolding each other with a series of juicy insults. Witnessing the outburst, the audience responded with hearty laughter. They laughed even more when Lomov, still cursing, left the part of the meadow considered the stage to lay down on the grass and returned with an imprint of fresh cow dung on his back. And when the group performed the same play again, Lomov’s new-found nonchalance was visible upon his meeting with Chubukov’s daughter and his new fiancée, Tatyana Stepanovna. Instead of the tent wing that played the table in Chubukov’s salon during the performance by the Neretva river, now that role was played by an old sewing machine. Lomov sat down next to it and began to turn the wheel of the machine by pushing the pedal with his foot. He asked the actress playing Stepanovna if she sewed and if she would be willing to make him a new tailcoat. When she answered that his frayed jacket, which most probably still bore the remains of cow dung, was so nice that he did not need another one, the audience responded with delighted laughter. In fact, it seems the audience was happy to reward all the actors’ outbursts with laughter and applause, making the somewhat unpolished performances nevertheless successful, often exceeding the expectations of the actors themselves.^61^

Just as the groups of peasants were happy to watch what the members of the theatre prepared for them, when the official part of the theatre programme ended, they continued to socialise with equal enthusiasm, mostly with song and dance. During one of the nights, there were also soldiers in Seona who had been commanded by Sava Kovačević before his death in June. The fighters were dispirited not only because of the tragic developments at Sutjeska, but also because of the loss of their commander. Kovačević, who was known among some comrades as the Montenegrin Chapayev,^62^ was seemingly universally liked, even loved, and had already become a legend in his own lifetime. Dancing a circle dance and singing a series of songs dedicated to him – surely one of them was the song about his famed charge against Italian tanks: ‘In the engine gasoline blazes/Standing on a tank Sava declares:/Charge, my brother Partisans/You made the Italians cut and run/Run toward the blue sea/Leave Montenegro free’^63^ – they said goodbye to him. Just a few months earlier – for instance, following the successful extraction from the Neretva valley and the liberation of the town of Nevesinje just a few days later – the same song ‘boomed’ as the soldiers and civilians sang and danced, as its words encouraged and inspired the soldiers.^64^ In this new situation, they served as a consolation for one of the thousands of dead left around Sutjeska.

## Conclusion

By considering both formal and informal settings of relaxation, socialisation, and having fun remembered by some of the members of the Theatre of the People’s Liberation, this article has offered an exploration of practices of socialisation and having fun among Yugoslav participants in the People’s Liberation Struggle. The group’s wartime pursuits were never focused solely on artistic expression; rather, as theatrologist Aldo Milohnić succinctly put it, through their work they aimed ‘to conceptualise radical performative-political practices’.^65^ The reason for this was, of course, that their ‘revolutionary artistry’ was inseparable from the development of antifascist resistance on the territory of Yugoslavia.^66^ Moreover, the group’s ability to adapt and perform in challenging circumstances demonstrated their commitment to the cause of the Partisan struggle. Therefore, the activities of the KNO went beyond the boundaries of traditional artistic creation, as was especially visible during the battles of Neretva and Sutjeska.

Through their wartime artistic endeavours, the members of the KNO, even in the most difficult moments, provided much-needed relaxation to the fighters during breaks between armed clashes with the enemy and to the civilians and the wounded who retreated with them. They entertained all of them and socialised and had fun with them, trying at the same time to maintain the fighters’ combat morale and stave off the terror surrounding all of them. In such moments, shared laughter, singing, and dancing played the most important role. As has been true in many wars before and since, this type of activity proved to be ‘unmatched in its power to cajole, console, cheer and inspire during the conflict and its aftermath’.^67^ The presence of the members of the Theatre of the People’s Liberation amid the chaos of war at both Neretva and Sutjeska inspired and uplifted both soldiers and civilians alike. At the same time, the actors fulfilled a social and political purpose, more apparent at some times than others. In other words, those who attended the group’s performances or joined in the singing around the nightly campfires enjoyed a unique amalgam of ‘a rally, an artistic performance, a political lesson, and a party’.^68^ Every new situation challenged the actors of the KNO to explore the transformative power of having fun in the face of adversity. Inhabiting, perhaps, the borderland where art spills into life, they provided respite and fun that, even in the worst of circumstances, resonated with their audiences.

## Footnotes

1 Vera Crvenčanin, *Ima tako ljudi …* (There are such people …) (Belgrade: Svijet knjige, 2012), 191. All translations in the article are the author’s.

2 Military forces led by the Communist Party of Yugoslavia held four different names during the war, some of them quite long. Early in the conflict, therefore, the term ‘Partisans’ became commonly used as their unofficial name. For that reason, when this term is capitalised, it refers specifically to Yugoslav armed resistance of the Second World War.

3 The battle of Neretva is locally also known as the Battle for the Wounded or the Fourth Enemy Offensive. Internationally it is known as the *Fall Weiss I* and *II*. Notably, the original Axis plan included a third part, *Fall Weiss III*, during which the forces would focus on the destruction of Chetnik units in eastern Herzegovina and Montenegro. Partly because of the circumstances on the ground, but also because of Italian opposition, it was cancelled in the first half of February.

4 The battle of Sutjeska is also known as the Fifth Enemy Offensive and, internationally, as the *Fall Schwarz*.

5 The People’s Liberation Struggle (*Narodnooslobodilačka borba*, NOB) is the name of the armed resistance of the so-called United People’s Liberation Front of Yugoslavia (*Ujedinjeni narodnooslobodilački front Jugoslavije*, JNOF) and the Partisan military units against foreign occupiers and their collaborators in the territory of Yugoslavia during the Second World War (1941–5). The Communist Party of Yugoslavia (*Komunistička partija Jugoslavije*, KPJ), led by Josip Broz Tito, played a leading role.

6 Ben Shepherd, ‘With the Devil in Titoland: A Wehrmacht Anti-Partisan Division in Bosnia-Herzegovina, 1943’, *War in History* 16, no. 1 (2009), 77–97; idem, *Terror in the Balkans: German Armies and Partisan Warfare* (Cambridge, MA: Harvard University Press, 2012).

7 Prior to September 1943, Italy occupied parts of the Yugoslav lands and fought against the Partisans. The Independent State of Croatia (*Nezavisna država Hrvatska*, NDH) as well as each of the occupied units established in other Yugoslav territories had some form of military force. In addition, another guerrilla army, the Chetniks, operated in most parts.

8 Slavko Goldstein and Ivo Goldstein, *Tito* (Zagreb: Profil, 2015), 270, 278.

9 Aldo Milohnić, *Gledališče upora* (Theatre of rebellion) (Ljubljana: Akademija za gledališče, radio, film in televizijo Univerze v Ljubljani and Znanstvena založba Filozofske fakultete Univerze v Ljubljani, 2021), 73.

10 The Theatre of the People’s Liberation existed from April 1942 to November 1944. It was the most famous wartime theatrical group in the territory of Yugoslavia whose core consisted of seven members of the State Theatre in Zagreb (Independent State of Croatia). This theatrical group was named by and placed under the administration of the Antifascist Council of the People’s Liberation of Yugoslavia (*Antifašističko vijeće narodnog oslobođenja Jugoslavije*, AVNOJ), the general political representative body of the resistance. Most members of the group arrived in liberated Belgrade on 1 November 1944 (the city had been liberated eight days earlier). They ended their wartime journey with their last performance as the KNO on 12 November. Its members continued their professional careers in both theatre and film.

11 A great deal of literature about most aspects of the People’s Liberation Struggle was published during the existence of socialist Yugoslavia. Historiography is only a small part of the excessive creation and publication of content on the subject; narratives about the antifascist resistance and the accompanying revolution can be found in every type and genre of cultural production.

12 Paul Eakin, *How Our Lives Become Stories: Making Selves* (Ithaca, NY: Cornell University Press, 1999).

13 Ben Fincham, *The Sociology of Fun* (London: Palgrave Macmillan, 2016), 19.

14 Vladimir Anić, *Rječnik hrvatskoga jezika* (Dictionary of the Croatian language), 3^rd^ edn (Zagreb: Novi liber, 1998), 1325.

15 Ana Hofman, *Novi život partizanskih pesama* (New life of the Partisan songs) (Belgrade: XX vek, 2016), 17.

16 Matej Bor, ‘Na pragu nove slovenske literature’ (On the threshold of the new Slovenian literature), *Setev* 1 (Summer 1943), 13.

17 Quoted in Miklavž Komelj, *Kako misliti partizansko umetnost?* (How to think Partisan art?*)* (Ljubljana: Založba /*cf, 2009), 284. This section of the landmark book on the Partisan art has been translated into English: Miklavž Komelj, ‘Contemporary Art: A Meeting of the Current and the Cosmic!’, in *Partisans in Yugoslavia: Literature, Film and Visual Culture*, edited by Miranda Jakiša and Nikica Gilić (Bielefeld: transcript, 2015), 31–48.

18 Komelj, *Kako misliti partizansko umetnost?*, 284.

19 Ibid., 292.

20 Iva Jelušić, ‘“The Mood in General Was Like At a Football Match’: Entertainment and Fun in the People’s Liberation Struggle’, *Prispevki za novejšo zgodovino* (forthcoming).

21 The Allies, however, decided to invade Italy and began their advance along the Apennine Peninsula in July 1943.

22 Stevan K. Pavlowitch, *Hitler’s New Disorder: The Second World War in Yugoslavia* (New York: Columbia University Press, 2008), 152.

23 Shepherd, ‘With the Devil’, 78.

24 In this liberated area, which at the end of 1942 occupied approximately 45,000 square kilometres, a civilian administration – including the first session of AVNOJ – was established with its centre in the Bosnian town of Bihać. It was referred to as the Republic of Bihać, while the Germans called it Tito’s State (*Titostaat*). Importantly, here and in other liberated territories during the war, the communist leadership of the resistance organised, for some people their first, theatre plays, but also medical centres, schools, courts, and parliamentary assemblies: Gregor Kranjc, ‘Collaboration, Resistance and Liberation in the Balkans, 1941–1945’, in *The Cambridge History of the Second World War,* *Volume II:* *Politics and Ideology*, edited by Richard J.B. Bosworth and Joseph A. Maiolo (Cambridge: Cambridge University Press, 2015), 466.

25 The Chetniks (formally the Chetnik Detachments of the Yugoslav Army) were a Serbian royalist and extreme nationalist movement and guerrilla force active in the territory of Yugoslavia during the Second World War. In its long-term goals, the Chetniks were an anti-Axis force, but they regularly engaged in collaboration with the Axis countries, primarily Italy.

26 According to the estimates, there were up to 150,000 Partisans in the entire Yugoslav territory (mostly in Bosnia and Herzegovina, Croatia, and Slovenia) at the time: Dušan Bilandžić, *Hrvatska moderna povijest* (Croatian modern history) (Zagreb: Golden marketing, 1999), 136–7.

27 Pavlowitch, 154; Shepherd, ‘With the Devil’, 84–5.

28 For Neapolitan officer Armando Mafrici, who participated in the offensive, Bosnia was ‘cold, with a fine air, then only woods, woods, woods’: quoted in Davide Rodogno, ‘Italian Soldiers in the Balkans: The Experience of the Occupation (1941–1943)’, *Journal of Southern Europe and the Balkans* 6, no. 2 (August 2004), 135, <https://doi.org/10.1080/1461319042000242029>

29 The term ‘Partisan colourful programme’ derives from the variety of content that theatre groups prepared and performed: Vjekoslav Afrić, ‘Sjećanje na KNO’ (Remembering the KNO), in *Hrvatsko narodno kazalište: zbornik o stogodišnjici (1860–1960)* (Croatian National Theatre: Anthology about the centenary (1860–1960)), edited by Duško Roksandić and Slavko Batušić (Zagreb: Naprijed, 1960), 114–23; Jelušić, ‘“The Mood In General”.

30 Nikola Hercigonja, ‘Partizansko kazalište’ (Partisan theatre), *Kulturni radnik* 7–8 (July–August 1955), 307.

31 Ibid.

32 Braslav Borozan, ‘Avangardno i inovacije u kazalištu NOB-a’ (Avant-garde and innovations in the NOB theatre), *Dani Hvarskoga kazališta: Građa i rasprave o hrvatskoj književnosti i kazalištu* 10, no. 1 (1983), 45.

33 Nikola Hercigonja, untitled text, in *Muzika i muzičari u NOB, zbornik sjećanja* (Music and musicians in the NOB, collection of memories), edited by Andrija Tomašek (Belgrade: Savez organizacija kompozitora Jugoslavije etc., 1982), 165–66.

34 Joža Rutić, ‘Sjećanje na Kazalište narodnog oslobođenja Jugoslavije’ (Remembering the Theatre of the People’s Liberation of Yugoslavia), *Kulturni radnik* 7–8 (July–August 1952), 332.

35 Đuka Kosak, ‘Introduction’, *Partizanska pozornica* (The Partisan stage) (Zagreb: Nakladni zavod Hrvatske, 1946), 10.

36 Zala Dobovšek, ‘Gledališče “za ceno življenja” v obleganem Sarajevu’ (Theatre ‘at the cost of life’ in besieged Sarajevo), *Dialogi: revija za kulturo in družbo* 51, no. 1–2 (January–February 2015), special issue: *Gledališče upora* (Theatre of rebellion), 138–43. Similar points on the role of theatrical activity, particularly in the introduction and in testimonies of several theatre artists who experienced the wars of the 1990s in the war zones of Croatia and Bosnia, are presented in: Jana Dolečki, Senad Halilbašić, and Stefan Hulfeld, eds., *Theatre in the Context of the Yugoslav Wars* (Cham: Palgrave Macmillan, 2018).

37 As quoted in Dobovšek, 140.

38 Sarajevo Film Festival. Dir. Johan van der Keuken, Lucid Eyefilms, 1993.

39 Borozan, 46.

40 Goldstein and Goldstein, 269.

41 Silvije Bombardelli, untitled text, in Tomašek, 44.

42 Branko Petranović, *Historija Jugoslavije (1918–1988), II knjiga: Narodnooslobodilački rat i revolucija (1941–1945)* (History of Yugoslavia (1918–1988), book 2: People’s Liberation War and revolution (1941–1945)) (Belgrade: Nolit, 1988), 252–3.

43 As quoted in Gaj Trifković, ‘A Case of Failed Counter-Insurgency: Anti-Partisan Operations in Yugoslavia 1943’, *Journal of Slavic Military Studies* 24 (2011), 330, <https://doi.org/10.1080/13518046.2011.572733>

44 Goldstein and Goldstein, 272–3.

45 Vjekoslav Afrić, ‘Pokret, vatra … i smrt’ (Advance, fire … and death), in *Sutjeska, zbornik radova 1* (Sutjeska, collection of works 1), edited by Milinko Đurović (Belgrade: Vojnoizdavački zavod JNA ‘Vojno delo’, 1958), 48.

46 Ibid.

47 The text for the song was written during the People’s Liberation Struggle. The melody, however, was appropriated from the Soviet *Partisan’s Song*, which in turn has its roots in a song written at the beginning of the First World War for the recruitment of soldiers into the army of Imperial Russia. This song is an example of the revolutionary songs, some international and some written by domestic authors, that Yugoslav communist activists, especially students and workers who were active during the interwar period as well as the volunteers in the Spanish Civil War (1936–39), sang. In other words, musical trends during the NOB were a continuation of the already existing trend of the pre-war musical left: Silvije Bombardelli, ‘Prilozi izučavanju muzike u NOB’ (Contributions for the study of music of the NOB) and Ante Nazor, ‘Borbene i reolvucionarne pjesme (Dalmacija 1941–1945)’ (Combat and revolutionary songs (Dalmatia 1941–1945)), both in *Mogućnosti* 28, no. 8–10 (1981), special issue: *Kultura u NOB-i u Dalmaciji – prilozi, građa, dokumenti* (Culture during the NOB in Dalmatia – texts, materials, documents), 919–38, 955–75.

48 ‘*Crne horde nas ne plaše,*/*Krv herojska u nas vri,*/*Mi ne damo zemlje naše*/*Da je gaze fašisti!*’Joža Rutić, ‘Osmi jun 1943 godine’ (The 8 of June 1943), in Đurović, 498.

49 Hercigonja, untitled text, 170.

50 Emma Hanna, ‘Say It with Music: Combat, Courage and Identity in the Songs of the RFC/RAF, 1914 − 1918’, *British Journal of Military History* 4, no. 2 (2018), 118.

51 Hercigonja, Untitled text, 164.

52 Ibid, 169.

53 Rutić, 498.

54 Graham Seal, ‘“We’re Here Because We’re Here”: Trench Culture of the Great War’, *Folklore* 124, no. 2 (August 2013), 190. Similar points were raised in: Tim Cook, ‘The Singing War: Canadian Soldiers’ Songs of the Great War’, *American Review of Canadian Studies* 39, no. 3 (September 2009), 224–41; Hanna, ‘Say It with Music’, 91–120; idem, *Sounds of War: Music in the British Armed Forces during the Great War* (Cambridge: Cambridge University Press, 2020).

55 Konstantin-Koča Popović, divisional commander of the First Proletarian Division, temporarily disbanded all auxiliary and technical units of the division and ordered that everyone, from officers to cooks, participate in the attack. Author Antonije Isaković described the charge: ‘We burst out of that forest and just ran, ran unstoppably … And our constant cry, more like a scream, from all of us: “Charge, proletarians! Forward! …” Just forward … We know we have nowhere to go but – forward. We must have looked terrible, from that forest, hairy and unshaven, starving, torn and battered … You just run, and you can see that yellow trench better and better; the Germans lying there, their *šarci* [MG42 machine guns] … The Germans are shooting, but we are advancing; one German, I see, gets up to run away. I remember that image: a German officer shooting him with a pistol, shooting at his soldiers. Does not let them run away. In vain, we defeated the Germans, I do not even know how. […] It seemed that we were going to slaughter them with our teeth.’: Boro Krivokapić, *Bes/konačni Tito (i Krležine ‘masne laži’)* (In/finite Tito (and Krleža’s ‘fat lies’)) (Belgrade: Novosti, 2006), 468.

56 Trifković, 322.

57 Žorž Skrigin, *War and Stage* (Belgrade: Turistička štampa, 1968), 139.

58 In the autumn of 1943, following Italy’s capitulation and particularly following Tito’s announcement that the Second Session of the AVNOJ would be held in November (held on 29 November), this city became the centre of the Yugoslav resistance forces. Bilandžić, 143–7.

59 Mira Banjanin, ‘S muzikom Druge proleterske divizije od juna 1943. do novembra 1944’ (With the music of the Second Proletarian Division from June 1943 until November 1944), *Mogućnosti* 28, no. 8–10 (1981), special issue: *Kultura u NOB-i u Dalmaciji – prilozi, građa, dokumenti* (Culture during the NOB in Dalmatia – texts, materials, documents), 939.

60 Skrigin, 215.

61 Hercigonja, untitled text, 170–73.

62 Quoted in Ivo Matović, *Sava Kovačević: Crveno znamenje Sutjeske* (Sava Kovačević: red signs of Sutjeska) (Donji Milanovac: Dečje novine, 1973), 73.

63 ‘*U motoru benzin gori,*/*a sa tenka Sava zbori;/Juriš, braćo partizani,*/*pobjegoše Talijani,*/*pobjegoše sinjem moru,*/*ostaviše Crnu Goru* […].’: Nikola Hercigonja and Đorđe Karaklajić, ‘Foreword’, *Zbornik partizanskih narodnih napeva* (Collection of the Partisan folk songs) (Belgrade: Nolit, 1962), xiv.

64 Ibid, xv. It is worth mentioning here that, along with the already mentioned tradition of revolutionary songs, folk songs were the main musical background for the activities of the Partisans and their supporters. For these people, who were mostly of peasant origin, it was often difficult to learn songs that went beyond traditional ways of singing, primarily because of new and unfamiliar melodies. It seems they best enjoyed and had the most fun with traditional folk music: Maja Bošković-Stulli, *Petokraka, zašto si crvena: narodne pjesme iz ustanka* (Five-pointed star, why are you red: folk poems of the uprising) (Zagreb: Lykos, 1959); idem, ‘Narodna poezija naše oslobodilačke borbe kao problem savremenog folklornog stvaralaštva’ (Folk poetry of our liberation struggle as a matter of contemporary folklore creation), *Glasnik Etnografskog instituta Srpske akademije nauka* 68 (1960), 393–424.

65 Aldo Milohnić, ‘Gledališče upora, uvod v temo’ (Theatre of rebellion, an introduction to the topic), *Dialogi: revija za kulturo in družbo* 51, no. 1–2 (January–February 2015), special issue: *Gledališče upora* (Theatre of rebellion), 6.

66 M. Hribar-Ožegović, ‘Kazališna djelatnost u Jugoslaviji za vrijeme NOB-a’ (Theatrical activity in Yugoslavia during the NOB) (PhD dissertation, Filozofski fakultet u Zagrebu, 1965), 5.

67 Hanna, ‘Say It with Music’, 120.

68 Hercigonja, Untitled text, 170.

